# Acute Laryngitis

**Published:** 1853-01

**Authors:** Wm. H. White


### ART. IV.—*Acute Laryngitis.* By Wm. H. White, M. D.

In the November number of your Journal I read a very interesting case of acute laryngitis, upon the subject of which, tracheotomy was successfully performed, while the patient was apparently in *articulo mortis.* I propose to give you the history and treatment of a case which occurred in my practice. I do not, however, expect it to be of equal interest as it was not necessary to perform tracheotomy, and therefore the *eclat* which hangs around *this*, as well as all successful surgical operations, is lost. Eminent surgeons have wisely said that it was more creditable to save a limb than to successfully amputate it. Viewed in this light my case may meet with some approbation, yet this is not my object in sending it to you. It is in the hope that it may be of benefit and interest to members of the profession, and call forth from them (as the above case did from me) cases and remarks of a similar nature.

*Aug.* 17, 1850. Mr. James McG——, an Irishman, between fifty-five and sixty years of age, called at my residence about 8 o'clock, A. M., (in a drizzling rain storm) complaining of a violent pain in the left cartilage of the pomum adami, which was increased upon pressure, with some considerable dyspnœa and difficulty of deglutition. He informed me that he had not been able to sleep the night previous, and could only lie on the right side: if he turned on the left side, or on the back, he smothered, and especially so while on the former. His voice was husky and much below its ordinary pitch, so much so, that had I trusted to it alone I should have presumed him to be laboring under an attack of acute bronchitis. The countenance was flushed, the pulse hard, the skin corresponded on touch to its appearance, it being red, dry and hot. On examining the fauces, uvula and velum, I found what is that? I suppose the left side of the thyroid cartilage is intended them of a bright scarlet color, but no swelling either in them or the tonsils. I gave him calomel, grs. xxv, jalap, grs. x, to be taken as soon as he reached home (it being one mile distant) to be followed in two hours by sulp. magnesiæ, ℥i. I saw him in half an hour or so after, and took xx℥ of blood and applied *immediately* a blister to the side and posterior part of the neck and a solution of N. silver, grs. xl, aqua pura, ℥i, I applied with a probang to the larynx and fauces, and in my absence he gargled his throat with a strong solution of acetum and chloride of sodium. I visited him six times in course of the day, and each time applied N. silver. At 11 o'clock, on visiting him. I found the cathartic had operated well; I then put him on digitalis, colchicum and calomel A. A. grs. i, every two hours. The blister having drawn, I dressed it with ung. hydrarg.

*Aug.* 18, 4 o'clock in the morning, was called in haste to see Mr. McG. Found he had experienced great dyspnœa for the past three or four hours, was exceeding restless, and his countenance betokened the utmost imaginable fear and trouble of mind, and by the most excited actions urged to have the windows and door thrown open. I immediately applied the probang moistened with N. silver to the larynx when considerable — thickened mucus was expelled and relief to some extent was experienced. The fauces at this time did not appear so congested as the day previous. On inquiry I found that in the early part of the night the bad symptoms seemed to be mitigated. The blister was again dressed with ung. hyd. and a wash for the throat and larynx made of the chlorate of potash ʒi, aqua pura, ℥ii, which was to be applied alternate every two hours with the N. S. The powder given the day before was continued.

10 o'clock. Patient somewhat easier save from pain at the neck of the bladder and in the testicle and cord corresponding to the cartilage of the larynx which was first complained of. For the new complication I gave a strong decoction of uva ursi and buchu, and had hops, soaked in hot water, placed over the bladder and testicle.

1 o'clock. Pain in bladder and testicle easier, otherwise no marked alteration. 4 o'clock. Pulse 86 and soft, not much fever, breathing easier, not as restless, but increased difficulty of swallowing, so much so that he thought it impossible to swallow the powders. I ordered injections of tinct. digitalis every four hours; if there should arise much febrile symptoms, every three hours. The application of the potash and silver continued every four hours.

*Aug.* 19*th*, 6 o'clock, A. M. Breathing better, voice quite clear, (which had been entirely lost,) can lie on either side; deglutition also better. Treatment: Injections of chicken broth with digitalis and colchicum every six hours, and chlorate of potash to the throat continued. 12 o'clock. The mercury has had its specific effect. Calomel and ung. hydg. discontinued. The chicken broth, with the digitalis, continued every four hours.

*Aug. 20th.* Patient was quite comfortable.

*Aug. 24th.* Mr. McG. thinks he is well. Appetite good. Advised him not to take much food and no stimulating drinks. He has long been in the habit of taking intoxicating drink to excess.

*Remarks.* — I bled my patient but once, as I believe one general bleeding from 12 to 20℥, according to the habit and constitution of the patient, is all that is required in any inflammation. This class of diseases may often be temporarily relieved by bleeding at short intervals, as is by some recommended, but in my opinion it tends to leave the system in a state unable to perform a cure from a want of sufficient power in the active organs of life to rally when the main disease is subdued.

I blistered early. My experience has taught me its correctness. The counter-irritation and discharge from the denuded surface, has a direct and beneficial influence upon the seat of the disease, by causing the morbid excitement centered at the diseased point to be turned toward it, and thus a decided blow is at once struck. Most authors, and I presume most practitioners, judging from those I have met in council, &c., are opposed to this course in any inflammation, and especially directly over, or near the larynx, when it is inflamed; for fear of increasing the unfavorable symptoms by causing increased serous infillration in the cartilage and glottis. Prof. Watson, however, says that "it is possible the œdema of the glottis might be produced or augmented in consequence of these topical remedies," (referring also to local bleeding.) From this there would seem to be a strong doubt in his mind as to its having this effect. It appears to me that the capillary vessels of the glottis are too *far off* to be materially affected by any external irritant.

I think the blister, N. silver and chlorate of potash, were the main remedies in effecting the cure.

